# A Prospective Study of the Medication Regimen Complexity Index and Hospitalization Due to Adverse Drug Reactions Among People Living with HIV

**DOI:** 10.3390/medicina60101705

**Published:** 2024-10-17

**Authors:** Basavaraj Poojar, Ashwin Kamath, Sathish B. Rao, Sheetal Dinkar Ullal, John Ramapuram, Muralidhar B. Yadiyal, Ashok K. Shenoy

**Affiliations:** 1 Department of Pharmacology, Kasturba Medical College Mangalore, Manipal Academy of Higher Education, Manipal 576104, Karnataka, India; basavaraj.poojar@manipal.edu (B.P.); ashwin.kamath@manipal.edu (A.K.); sheetal.ullal@manipal.edu (S.D.U.); 2 Department of General Medicine, Kasturba Medical College Mangalore, Manipal Academy of Higher Education, Manipal 576104, Karnataka, India; sathish.rao@manipal.edu (S.B.R.); yadidal.m@manipal.edu (M.B.Y.)

**Keywords:** medication regimen complexity index (MRCI), adverse drug reactions (ADRs), hospitalization, people living with HIV (PLHIV), antiretroviral therapy (ART)

## Abstract

*Background and Objective*: The complexity of antiretroviral therapy (ART) regimens in people living with HIV (PLHIV) poses significant challenges for medication management, impacting adherence and overall health outcomes. The Medication Regimen Complexity Index (MRCI) is a tool that quantifies regimen complexity, yet its correlation with hospitalization rates and adverse drug reactions (ADRs) in PLHIV remains underexplored. *Materials and Methods*: This prospective study, which was conducted at a government-funded antiretroviral treatment center, investigated the relationships among MRCI scores, hospitalization due to ADRs, and the ADR rates in 285 PLHIV participants over 18 months. *Results*: The study revealed a significant association between higher baseline MRCI scores and hospitalization due to ADRs, with a threshold MRCI score of 8 indicating increased risk. There was no significant association between average MRCI scores and overall ADR rates or non-ADR-related hospitalizations. *Conclusions*: These findings emphasize the importance of monitoring medication regimen complexity in PLHIV, particularly in the context of preventing hospitalizations related to ADRs. Further research is needed to understand the multifactorial influences on ADR occurrence and to optimize ART regimens for better patient outcomes.

## 1. Introduction

Medication management in people living with HIV (PLHIV) presents unique challenges due to the complexity of antiretroviral therapy (ART) and the potential for adverse drug reactions (ADRs). As PLHIV are required to adhere to lifelong medication regimens, understanding the factors influencing medication management and its impact on health outcomes is crucial for optimizing care and improving patient outcomes. One such factor of interest is the Medication Regimen Complexity Index (MRCI), a quantitative measure used to assess the complexity of medication regimens [1].

The MRCI is a validated tool developed to quantify the complexity of medication regimens on the basis of various factors, such as dosage forms, dosing frequency, and administration instructions [1]. Higher MRCI scores indicate greater regimen complexity, which may pose challenges to medication adherence and management. Previous research has demonstrated associations between high MRCI scores and suboptimal adherence, increased healthcare utilization, and poorer clinical outcomes in various patient populations [2,3,4]. In the context of PLHIV, optimizing medication management is essential for achieving viral suppression, preserving immune function, and reducing the risk of disease progression and transmission. However, the relationship between the MRCI and health outcomes in PLHIV remains underexplored. Specifically, there is limited research examining the association between MRCI and hospitalization rates as well as the occurrence of ADRs in PLHIV.

Hospitalization rates serve as critical indicators of disease severity, treatment effectiveness, and overall healthcare utilization in PLHIV [5]. High hospitalization rates not only impose significant burdens on healthcare systems but also reflect suboptimal disease management and outcomes [6]. Understanding the factors contributing to hospitalization in PLHIV, including medication regimen complexity, can inform targeted interventions to reduce hospitalization rates and improve patient care. Similarly, ADRs represent a significant concern in the management of PLHIV, as ART regimens are associated with a range of potential side effects and toxicities [7]. ADRs not only impact patient well-being and quality of life but also may result in treatment interruptions, regimen switches, and increased healthcare utilization. Exploring the relationship between MRCI and the occurrence of ADRs in PLHIV can provide insights into the safety and tolerability of ART regimens and guide clinical decision-making.

Therefore, the primary objective of this study was to determine the association of MRCI in PLHIV with hospitalization rates and the number of ADRs. By investigating these associations, we aim to identify potential modifiable factors influencing health outcomes in PLHIV and inform strategies to optimize medication management and improve patient care.

## 2. Materials and Methods

This prospective study was conducted at the government-funded antiretroviral treatment center of Kasturba Medical College Hospital (KMCH), located in Attavar, Mangaluru. The center serves the population of the Dakshina Kannada district in the southern part of Karnataka and provides clinical services to patients from adjacent districts and the neighboring state of Kerala. The study commenced with the approval of the Institutional Ethics Committee of Kasturba Medical College, Mangaluru, Karnataka, India (IEC KMC MLR 05–19/227). The study was registered with the Clinical Trials Registry—India (CTRI) under registration number CTRI/2019/06/019609. The inclusion and exclusion criteria and the sample size calculation have been described in an earlier study [8].

### 2.1. Assessment of the MRCI

The MRCI is a validated instrument designed to quantify the complexity of medication prescriptions [1]. The MRCI consists of three distinct sections: A, B, and C. Section A evaluates the pharmaceutical formulation of the drug; Section B assesses the frequency of drug administration; and Section C examines the specific instructions for medication administration. Each section is scored individually, and the cumulative score of these three sections represents the total MRCI score. The baseline and average MRCI scores over the study duration were determined.

### 2.2. Hospitalization

The frequency of hospitalizations was determined at the conclusion of an eighteen-month follow-up period. For each hospitalization event, the length of stay (LOS), the primary suspected diagnosis leading to hospital admission, and the MRCI scores were documented.

### 2.3. ADR Recording

For patient-reported ADRs (pADRs), information was obtained by querying patients regarding the presence of any adverse symptoms or discomfort experienced subsequent to commencing ART. The queries were about collective ART-related ADR signs and symptoms identified on the basis of earlier published literature [9,10,11,12,13] and WHO treatment guidelines [14]. The WHO guidelines listed the most common ADRs associated with ART, which we determined to be relevant for patient reporting. Accordingly, we included these commonly reported ADRs in our survey, alongside symptoms identified in the earlier literature, to comprehensively elicit information from patients. The respondents were asked whether they had experienced any of the following signs and symptoms: headache, nausea, skin rash or itching, diarrhea/loose stool, tingling of feet/hands or burning sensations, lethargy/fatigue, nightmare/bad dreams, or sleeplessness. In addition, the patients were asked to report any other ADRs experienced over the course of treatment. For weight gain, patients were asked if they had experienced any weight changes compared to their previous visit, with weight gain or loss categorized as a mean weight difference of 6 kg. For hyperglycemia, patients were asked about common symptoms such as frequent urination, excessive thirst, fatigue, increased hunger, dry mouth, and unintended weight loss. Additionally, other patient-reported ADRs were explored by asking patients about any joint pain, body aches, or related discomfort. An outpatient and inpatient medical record review was performed to collect details related to ART and concomitant medication(s) and previous and current laboratory investigation data.

The Division of AIDS (DAIDS) adverse drug reaction severity scale 2.1 was used to grade ADR intensity, ranging from Grade 1 (mild) to Grade 5 (death) [15]. ADR outcomes were classified according to the National Coordination Centre, Pharmacovigilance Programme of India standards: recovered, recovering, not recovered, fatal, recovered with sequelae, and unknown. Serious ADRs included those resulting in death, life-threatening events, hospitalization, congenital anomalies, and disabilities. Causality was assessed via the WHO-UMC causality assessment scale. Causality was classified as certain, probable, possible, unlikely, conditional, or unassessable. The Schumock and Thornton criteria, consisting of ten yes/no questions, were used to determine ADR preventability [16]. ADRs were categorized as definitely preventable, probably preventable, or nonpreventable on the basis of the responses across three sections. To determine predictability, the ADRs were classified into six subtypes on the basis of time and dose dependency via the Aronson method, [17,18] arranged alphabetically from A to F.

WHO-UMC Causality Scale: The WHO-UMC causality scale was utilized to assess the likelihood of a causal relationship between the suspected drugs and adverse drug reactions (ADRs). This scale categorizes causality into “certain”, “probable”, “possible”, etc., based on clinical and temporal associations. For example, an ADR occurring shortly after drug intake, unlikely to be due to the disease itself or other drugs, and resolving upon withdrawal would be considered “probable”.

DAIDS Adverse Event Scale: The DAIDS adverse event scale was employed to grade the severity of ADRs, ranging from Grade 1 (mild) to Grade 5 (fatal). For example, a liver enzyme increase of 1.1–2.5 times the upper limit of normal is classified as Grade 1, whereas an increase of fivefold or more is categorized as Grade 4 (severe). The DAIDS scale is specifically designed for HIV clinical trials and was particularly relevant for assessing ADRs in our population of people living with HIV (PLHIV).

Ensuring Consistency Among Raters: To maintain consistency in ADR assessments, the same primary rater assessed all the reported ADRs. In addition, 10% of the reported ADRs were also assessed by another investigator to check for accuracy of the rating. Any difference in assessment was resolved by consensus.

### 2.4. Statistical Analysis

The data were entered twice via EpiData Entry software version 3.1 (EpiData Association, Odense, Denmark) and analyzed via IBM^®^ SPSS^®^, version 11.0 (Chicago, IL, USA). The normality of the data distribution was assessed via the Shapiro–Wilk test; the study parameters were not normally distributed (*p* < 0.05). The MRCI and ADR data are presented as frequencies/percentages or medians and interquartile ranges, as appropriate. Study groups were compared using the Mann–Whitney U test. The correlation between the MRCI score and the number of ADRs was assessed via Spearman’s correlation coefficient test. The chi-square test was used to analyze the associations between categorical variables (MRCI and hospitalization). A *p* value < 0.05 was considered to indicate statistical significance.

## 3. Results

### 3.1. Demographic Analysis

**Figure 1:**
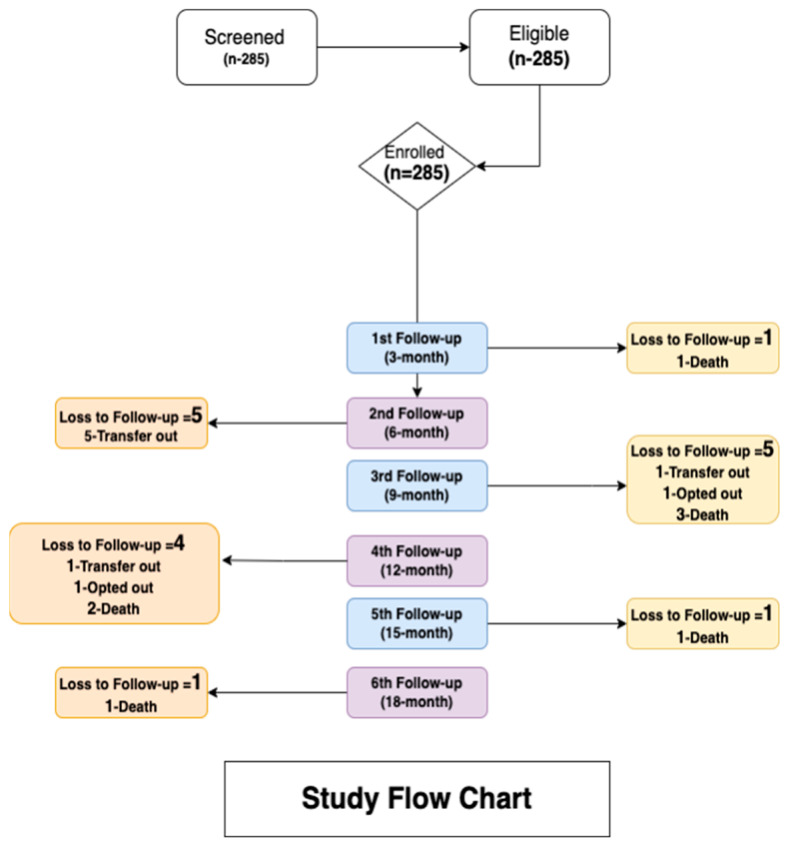
Study flow chart.

Two hundred and eighty-five participants were screened Figure 1. All participants were eligible and were enrolled in the study. Two hundred and sixty-eight participants completed the 18-month study period. The median age of the study participants was 48 years (interquartile range [IQR], 39.50–54.00); 147 (51.6%) were males, and 138 (48.4%) were females. The demographic characteristics of the study sample have been described in an earlier study. Furthermore, Table 1 outlines the clinical and disease-related characteristics of the PLHIV population under investigation.

Of the antiretroviral regimens prescribed over the study duration, Tenofovir + lamivudine + efavirenz (TLE) was the most common regimen prescribed, accounting for 34.4% of the cases (n = 98). This was followed by tenofovir + lamivudine + dolutegravir (TLD) at 26.7% (n = 76) and tenofovir + lamivudine + atazanavir/ritonavir (TLATV/R) at 17.5% (n = 50). Collectively, these three regimens constituted 80% of the total prescribed, whereas the remaining thirteen regimens constituted the remaining 20%.

The sex distributions of the suspected drugs and ADRs are shown in Table 2 and Patient Reported adverse drug reactions are shown in Table 3. Fatigue was the most prevalent ADR (13.11%), followed by sleeplessness (12.84%) and weight gain (12.29%). With respect to the system organ class affected, nervous system disorders presented the highest prevalence (24.31%), followed by psychiatric disorders (23.49%) and musculoskeletal and connective tissue disorders (17.21%). The analysis of adverse events by System Organ Classes (SOCs) revealed that nervous system disorders were the most frequently reported, accounting for 24.31% (n = 89) of all cases. Psychiatric disorders followed closely at 23.49% (n = 86) of the participants. In total, 21.85% (n = 80) of the participants reported musculoskeletal and connective tissue disorders, whereas 17.21% (n = 63) reported musculoskeletal and connective tissue disorders. General disorders and administration site conditions were the least common, constituting 13.11% (n = 48) of the reported events. In total, 366 cases were documented across all SOCs.

In terms of the number of ADRs, 120 ADRs were recorded at baseline, peaked at 155 at the 3rd follow-up visit, and subsequently decreased to 144 by the 6th follow-up. On considering only the pADRs, a greater number of ADRs were reported by male patients (53.10%) compared to female patients (46.90%) (Z = −1.041, *p* = 0.298).

Out of a total of 915 ADRs, 505 (55.2%) were mild in severity, 322 (35.2%) were moderate in severity, and 74 (8.1%) were severe; 12 ADRs (1.3%) were classified as potential life-threatening events. Ten serious ADRs were recorded during the study period. In addition to serious ADRs, there were 63 episodes of hospitalization and five deaths that were judged not related to the prescribed medications. Regarding the preventability of ADRs, most of them were nonpreventable across all follow-ups. No significant difference in ADR preventability between males and females was observed at any follow-up visit. All the ADRs were of an augmented nature; 0.43% of the ADRs were considered to be preventable.

### 3.2. Association of MRCI in PLHIV with Hospitalization Due to ADRs

The baseline MRCI score (median [IQR]) was 8 (6.5–12). The MRCI scores remained fairly consistent throughout the study duration, with the average MRCI score over the study duration being 8 (4–12). Ten participants experienced an episode of hospitalization due to an ADR; seven of these were females (*p* = 0.165). The details of the ADRs that resulted in hospitalization are presented in Table 4. The baseline and average MRCI scores were significantly higher among those who were hospitalized due to ADR (Z = −2.328, *p* = 0.020; χ² = 6.233, df = 1, *p* = 0.013) than among those who were not hospitalized (Z = −0.725, *p* = 0.468;). Although more patients with an MRCI >8 experienced hospitalization due to ADR (χ² = 5.856, df = 1, *p* = 0.016), the median MRCI scores were not significantly different from those of those who were not hospitalized (Z = −0.725, *p* = 0.468).

### 3.3. Non-ADR-Related Hospitalization

Non-ADR-related hospitalization occurred in 21.80% of males (32/147) and 15.20% of females (21/138), and the difference was not statistically significant (*p* = 0.155). Overall, 18.60% (53/285) of the study participants experienced non-ADR-related hospitalization (Table 5 and Table 6). One hospitalization occurred among 45 patients (15.79%), 7 (2.45%) were hospitalized twice, and 1 (0.35%) was hospitalized thrice during the study period. The average MRCI score was not significantly different between those who were hospitalized and those who were not (8.00 [4.57–12.29] and 8.00 [5.14–12.00], respectively; Z = −0.450, *p* = 0.652). A significantly greater percentage of patients with an average MRCI >8 experienced hospitalization (30/122 [42.8%] vs. 23/163 [57.2%]; χ² = 5.062, df = 1, *p* = 0.024).

### 3.4. Correlation of the MRCI with the Number of ADRs among PLHIV

**Figure 2:**
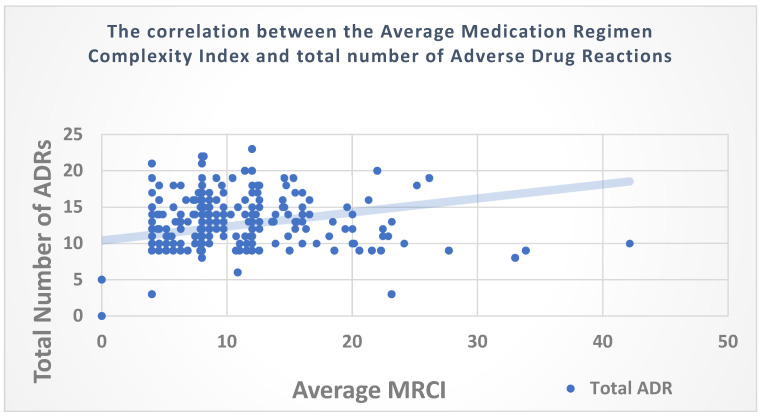
Scatter plot of the correlation between the average medication regimen complexity index and the total number of adverse drug reactions.

No significant correlation was observed between the baseline MRCI and the total number of ADRs (ρ = 0.074, *p* = 0.216); however, there was a significant correlation between the average MRCI score and the total number of ADRs (ρ = 0.263, *p* <0.001) (Figure 2). Correlations were also determined taking into account only the pADRs; no significant correlations were observed with the baseline MRCI (ρ = -0.005, *p* = 0.937) or average MRCI (ρ = 0.033, *p* = 0.588). No significant sex difference was detected in the total number of ADRs reported throughout the study duration (males, 12 [10,11,12,13,14,15]; females, 13 [10,11,12,13,14,15]; Z = −0.085, *p* = 0.932).

## 4. Discussion

In the present study, we investigated the relationship of medication regimen complexity with the number of ADRs reported and the hospitalization rates due to ADRs in 285 PLHIV. A significant association was observed between the baseline as well as average MRCI and hospitalization due to ADRs, indicating an increased likelihood of hospitalization in those receiving complex medication regimens. However, no association was detected between the average MRCI and non-ADR related hospitalizations or the number of ADRs.

In a study by Wimmer et al., the median MRCI was 9 (IQR, 4–16), with 38.4% of participants taking five or more medications. Over three years, 33.6% of the participants experienced unplanned hospitalizations, with higher MRCIs and medication counts significantly associated with hospitalization risk (HR 1.22; 95% CI 1.14–1.34 and HR 1.07; 95% CI 1.04–1.09, respectively). However, in fully adjusted models, these associations did not remain significant, suggesting that other variables may influence the risk [19]. Willson et al. [3], in their retrospective, parallel-group, case–control study across four urban acute care hospitals, reported a greater MRCI in patients readmitted for ADEs within 30 days than in those without readmission. Receiver operating characteristic curves were used to determine a potential MRCI cutoff score of 8 for predicting the risk of ADE-related readmission. Our study supports these findings, with a larger number of patients with MRCIs >8 being hospitalized due to ADRs.

Studies by Schoonover et al. [20] and Curtain et al. [21] revealed associations among high MRCIs, potential adverse drug events (ADEs), and unplanned hospital readmissions among patients transitioning from hospital to home care. Lepelley et al. [5] reported that higher MRCI scores upon admission were associated with increased odds of adverse outcomes during hospitalization [6,19].

In our longitudinal study, we observed varying durations of hospitalization (median 3–6.5 days) due to ADRs. Overall, the median duration of hospitalization was 4.5 (2.25–6.12) days. These findings align with those of the study conducted by Cortés et al. [22] investigating prior hospital admissions in patients with HIV infection. The results revealed a median hospital stay of 7 days (IQR, 4–12.5) among HIV patients, indicating the impact of hospitalization on medication regimen complexity (14.5 ± 7.2 before admission to 16.5 ± 8.0 after admission, with a mean difference of 1.97 (CI, 0.85–3.09)). This correlation emphasizes the importance of ongoing monitoring and management of ADRs to increase patient care, particularly within the context of HIV management, where hospitalizations could influence alterations in medication regimen complexity.

In our study, the MRCI score at baseline was 8 (IQR, 6.5–12), indicating the level of medication complexity at the beginning of the study. Various studies have reported MRCI scores ranging from 7 (IQR = 4–12) to 21.76 (±12.49) for PLHIV. An MRCI of 11.25 has been shown to be an indicator of polypharmacy (≥5 medications), with sensitivity and specificity values of 77.6% and 91.8%, respectively. It is evident from these studies that medication complexity is a significant concern, particularly in the management of HIV infection. Despite efforts to simplify treatment regimens, the evolution of HIV and the development of age-related comorbidities have contributed to an increase in the complexity of pharmacotherapy regimens for PLHIV over time. Studies among PLHIV with comorbidities such as diabetes, hypertension, and atrial fibrillation have shown the influence of comorbidities on increasing the regimen complexity [8,23]. However, in the present study, the MRCI scores remained consistent over the 18-month follow-up period. This stability in MRCI scores may be attributed to several factors. First, the stable disease condition of the participants may have contributed to the consistent medication complexity. Second, the median duration of ART being 6 years at the time of study entry may have influenced the stability of the MRCI scores. Individuals who have been on ART for an extended period may have already optimized their medication regimens, resulting in minimal changes in complexity over time. Furthermore, factors such as regimen adherence and medication tolerability may have also contributed to the consistent MRCI scores observed in our study.

Our study also examined the relationships between MRCI and the number of pADRs and total ADRs. No statistically significant correlations were observed, contrary to the expectation that an increase in MRCIs would result in more ADRs being reported. The possible reasons could include the complexity of factors influencing ADR occurrence beyond the complexity of the medication regimen alone, such as individual patient characteristics, medication interactions, and underlying health conditions. These nuances warrant further research to determine the intricate relationship between medication regimen complexity and ADRs comprehensively.

In our study, among the 10 patients hospitalized due to ADRs, seven cases were attributed to anemia. Anemia was also the most common ADR reported. The impact of anemia on patient quality of life and mortality cannot be understated. Previous studies have identified sex as a significant risk factor for anemia [24,25], which aligns with our findings that all seven ADR-related hospitalized patients were females. Anemia manifests with various clinical symptoms, including fatigue, weakness, dizziness, and drowsiness, significantly impairing patient quality of life [26,27]. In our study, fatigue was the most reported ADR among patients, accounting for 13.11% of the 366 reported ADRs, followed by weight loss at 9.56% and giddiness at 1.5%.

Recent evidence suggests that integrase strand transfer inhibitor (INSTI)-based regimens are associated with a heightened risk of anemia and severe anemia compared to non-nucleoside reverse transcriptase inhibitor (NNRTI)-based regimens. The adjusted hazard ratios for anemia and severe anemia were 1.26 (95% CI: 1.00 to 1.58) and 1.51 (95% CI: 1.07 to 2.11), respectively [28]. Furthermore, the proportion of patients on INSTI-based regimens increased significantly from 26.66% at baseline to 60.82% by the conclusion of our study, while the distribution of protease inhibitor (PI)-based regimens remained relatively stable, fluctuating between 21% and 22% throughout the study duration.

In our study, anemia was more common among females, with 49.27% (203 out of 412) of recorded ADRs in females attributed to anemia. This finding aligns with that of Manaye et al., who reported an overall incidence of anemia of 27 per 100 person-years [27]. Specifically, Manaye et al. noted the highest incidence of anemia in the second year (18.7 per 100 person-years) following ART initiation, compared with the first year (13.8 per 100 person-years) and third year (18.1 per 100 person-years). Additionally, independent predictors associated with the development of anemia included being female and having a baseline weight of less than 60 kg. In our study population, the median weight was 52 kg (IQR 49–64), which is consistent with the findings of Manaye et al. [27].

For the majority of FDA-approved drugs analyzed in one study, women exhibited elevated blood concentrations and prolonged elimination times, which were closely associated with sex differences in adverse drug reactions (ADRs). Out of the 86 drugs studied, 76 demonstrated higher pharmacokinetic (PK) values in women. Among the 59 drugs with clinically significant ADRs, sex-biased PK profiles predicted the direction of sex-biased ADRs in 88% of instances. Notably, 96% of drugs exhibiting female-biased PK values were linked to a greater occurrence of ADRs in women compared to men [29]. These gender differences in drug pharmacokinetics significantly contribute to increased drug toxicity in women. Such disparities arise from physiological variations, including body composition, plasma protein levels, and liver and kidney functionality, as well as drug interactions and comorbidities [30].

In our study, 176 participants (61.75%) self-reported ADRs (pADRs); 94 out of 147 males (63.94%) and 82 out of 138 females (59.42%) reported ADRs. This incidence contrasts with that reported by Tadesse et al., who reported a pADR prevalence of 89.8% over a three-month period [31]. However, the duration of ART treatment among the cohort was not specified. Notably, the most frequently reported ADRs in Tadesse’s study were nausea (56.5%) and headache (54.9%), which were not common in our study. Nausea typically occurs at the initiation of treatment; our population had a median ART duration of 6–9 years, suggesting potential tolerance or adaptation to treatment side effects. Despite these variations, our study demonstrated a comparable trend in ADR incidence over time. At baseline, out of 285 patients, 120 (42.10%) reported experiencing ADRs. During follow-up visits, the proportion of patients who experienced ADRs ranged from 43.01% to 56.77%, which aligns closely with the findings of Tadesse et al. [31].

The next common ADR observed in our study was an increase in creatinine levels, noted in 14.97% (137 out of 915 recorded ADRs), predominantly affecting males. The age range of the patients who experienced ADRs was between 41 and 64 years, and tenofovir disoproxil fumarate (TDF) was the suspected drug used in these patients. Specifically, 55.73% (510 out of 915 ADRs) of the recorded ADRs were attributed to TDF. According to the DAIDS severity grading scale, most ADRs are classified as mild. Additionally, blood urea elevation, which serves as an indicator of nephrotoxicity, is more commonly observed in males than in females, as highlighted by Agrawal et al. [32]. Their study suggested that an elevation in serum creatinine may indicate the onset of renal impairment, emphasizing the need for timely intervention to prevent further nephrotoxicity progression.

Research by Agbaji et al. [33] and Tan et al. [34]. reported that prolonged exposure to TDF increases the risk of renal function decline. Their findings revealed a significant increase in the prevalence of renal impairment among patients on TDF-based ART, with rates increasing from 13% at 48 weeks to 35% and 45% at 96 and 144 weeks, respectively. In contrast, patients not exposed to TDF had a relatively stable frequency of renal impairment at 14% after 144 weeks. Our study, with a median TDF exposure duration of 288–432 weeks, revealed a gradual increase in elevated creatinine levels over a 72-week follow-up period. Although proteinuria assessment was not initially recommended, current National AIDS Control Organization guidelines advise regular monitoring of urea and creatinine levels every three months for patients on DTG-based regimens containing TDF [35].

Shivakumar et al. [36] reported that continued exposure to TDF may impede complete recovery and lead to tubular disease and CKD with irreversible damage. Given the prevalent use of TDF-based regimens in resource-limited settings, where establishing screening and monitoring protocols is challenging, there is a critical need to reconsider this approach. It is essential to assess the serum creatinine level and estimated glomerular filtration rate (eGFR) before initiating TDF-containing ART alongside HIV testing and treatment initiation procedures.

The third most common ADR recorded in our study was dyslipidemia, accounting for 15.19% of the ADRs (139 out of 915 ADRs). This finding contrasts with previous studies conducted in Africa, where the prevalence of dyslipidemia ranged from 55% to 90% (328,552). However, lower prevalence rates were reported in studies conducted in China (32.2%) [37] and Iran (30.0%) [38]. These disparities may stem from variations in urbanization levels, socioeconomic statuses, dietary patterns, and levels of physical activity among study populations.

### Strengths and Limitations

This study has several notable strengths. First, a comprehensive analysis of ADRs through active surveillance of both clinical and laboratory parameters was performed. The prospective design of the study further strengthens its robustness by minimizing data loss, allowing for more accurate analysis and interpretation. Additionally, the inclusion of a large sample size and a relatively long follow-up period contributed to the thoroughness of the analysis and improved the generalizability of the results to broader populations.

However, there are also several limitations to consider. The study was conducted at a single urban center, which may limit the generalizability of the findings to other healthcare settings and patient populations. Furthermore, potential variability in MRCI scores could be a factor, as participants had been on ART for more than six months with an adherence rate exceeding 95%. This could have influenced the MRCI scores, particularly during the initial six-month period; therefore, the study findings may not be applicable to patient populations without good adherence to treatment.

## 5. Conclusions

Our analysis revealed a significant association between the baseline MRCI score and hospitalization due to ADRs, indicating the predictive role of medication complexity in adverse outcomes requiring hospitalization. The assessment of MRCI can serve as an additional tool for physicians, especially when initiating ART in newly diagnosed PLHIV or those experiencing comorbidities and treatment failure requiring a switch to second-line ART.

## Figures and Tables

**Table 1:** Clinical and disease-related characteristics of the PLHIV population.

Mode of Transmission		
Heterosexual intercourse	271	95.1%
Mother to Child	12	4.2%
Unknown	2	0.7%
Partner’s HIV status		
HIV-positive partners	126	44.2%
HIV-negative partners	61	21.4
Mother is-positive or Both parents are-positive	8	1.4%
Missing information (Unknown status)	90	31.6%
Family size		
3-Member Families	60	21.1%
4-Member Families	79	27.7%
>5-Member Families	35	12.4%
Not Recorded	43	15.1%
≤2 *	68	23.9%
Entry Point to healthcare system		
VCTC	168	58.94%
OPD	32	11.22%
Pvt Practitioner	78	27.36
PPTCT	3	1.5%
NGO	3	1.5%
IP	1	0.35%
Previous antiretroviral (ARV) usage prior to enrollment in the NACO Program		
Yes	105	36.8%
No	137	48.1%
Unspecified or Missing information	43	15.1%
World Health Organization (WHO) Clinical Stage at the initiation of ART	N	%
Stage-I	194	68.1%
Stage-II	18	6.3%
Stage-III	22	7.7%
Stage-IV	51	17.9%
History of TB	76	26.7%
PTB ETB	61 15	21.4%
TB Treatment Status		
		Ongoing
		Cure
		Completed
Baseline CD4 at the time of study enrollment (in cells/mm^3^)		
>500	145	50.9%
200–499	115	40.4%
<200	25	8.8%
>500	145	50.9%
End of the study CD4 (current status)		
>500	139	48.8%
200–499	102	35.8%
<200	18	6.3%
Missing	26	9.1%
Baseline HIV Viral Load (HIV RNA copies) at the time of study enrollment		
Target Not Detected	172	60.4%
Below 20 copies/mL	36	12.6%
>20–1,000,000 copies/mL	66	23.2%
Above 1,000,000 copies/mL	11	3.9%
End of the study Viral Load (HIV RNA copies)		
Target Not Detected	156	54.7%
Below 20 copies/mL	31	10.9%
>20–1,000,000 copies/mL	67	23.5%
Above 1,000,000 copies/mL	4	1.4%
Initiated regimen after the diagnosis of HIV infection		
Protease inhibitor-Based Regimen	5	1.75%
Nucleoside RT Inhibitors-Based Regimen	203	71.22%
Non-Nucleoside RT Inhibitors-Based Regimen	77	27.01%
Integrase Inhibitors-Based Regimen	0	0%
Regimen at the time of study enrollment (Baseline)		
Protease inhibitor-Based Regimen	62	21.75%
Nucleoside RT Inhibitors-Based Regimen	147	51.57%
Non-Nucleoside RT Inhibitors-Based Regimen	00.00	00.00
Integrase Inhibitors-Based Regimen	76	26.66%
First-line ART (at the time of study enrollment)	223	78.24%
Second-line ART (at the time of study enrollment)	62	21.75%

Voluntary Counseling and Testing for HIV (VCTC), Outpatient Department (OPD), Prevention of Parent-to-Child Transmission of HIV (PPTCT), Nongovernmental Organization (NGO), Inpatient (IP), Tuberculosis (TB), Pulmonary Tuberculosis (TB), Extrapulmonary Tuberculosis (EPTB); * Unmarried, Divorced.

**Table 2:** Gender distribution of adverse drug reactions and suspected medications.

Adverse Reaction	Male (n = 147)	Female (n = 138)	Total
Alanine aminotransferase increased	72	27	99
Aspartate aminotransferase increased	20	4	24
Hepatic enzyme increased (Combined AST and ALT)	45	6	51
Hyperbilirubinemia	20	21	41
Hypobilirubinemia	0	1	1
Blood alkaline phosphatase increased	1	9	10
Hyperamylasemia	2	0	2
Blood lactate dehydrogenase increased	2	0	2
Anemia	**106**	**203**	309
Neutropenia	3	4	7
Thrombocytopenia	7	15	22
Leukopenia	3	6	9
Dyslipidemia	**67**	**72**	139
Blood creatinine increased	**114**	**23**	137
Blood urea increased	19	6	25
Hyperglycemia	**21**	**13**	34
Hyponatremia	0	1	1
Hypersensitivity	0	1	1
Toxic optic neuropathy	1	0	1
	**503**	**412**	**915**
**Suspected Drugs**			
Abacavir/Lamivudine	13 (2.58%)	0	13 (1.42%)
Atazanavir/Ritonavir	**26 (5.16%)**	**28 (6.79%)**	**54 (5.90%)**
Dolutegravir	**67 (13.32%)**	**27 (6.55%)**	**94 (10.27%)**
Efavirenz	18 (3.57%)	7 (1.69%)	25 (2.73%)
Efavirenz/TLE	6 (1.19%)	3 (0.72%)	9 (0.98%)
Lamivudine	**86 (17.09%)**	**42 (10.19%)**	**128 (13.98%)**
Lamivudine/Abacavir	2 (0.39%)	0	2 (0.21%)
Nevirapine	1 (0.19%)	0	1 (0.10%)
Tenofovir	**234 (46.52%)**	**276 (66.99%)**	**510 (55.73%)**
Zidovudine	**32 (6.36%)**	**20 (4.85%)**	**52 (5.68%)**
Zidovudine/Lamivudine	14 (2.78%)	2 (0.48%)	16 (1.74%)
Ritonavir	3 (0.59%)	6 (1.45%)	9 (0.98%)
Amoxicillin	0	1 (0.24%)	1 (0.10%)
Ethambutol	1 (0.19%)	0	1 (0.10%)
	**503**	**412**	**915**

The four most frequent ADRs and the five most commonly implicated drugs are presented in bold.

**Table 3:** Patient Reported adverse drug reactions among 285 study participants with HIV infection.

	Frequency	Percent
Abnormal sensation of limbs	6	1.63%
Hypoesthesia	8	2.18%
Paresthesia	17	4.64%
Paresthesia generalized	14	3.81%
Paresthesia lower limb	11	3.00%
Skin hypoesthesia	4	1.09%
Anxiety state, unspecified	**32**	**8.74%**
Fatigue	**48**	**13.11%**
Intermittent headache	11	3.00%
Muscle contraction headache	3	0.81%
Headache	3	0.81%
Headache transient	4	1.09%
Throbbing headache	2	0.54%
Muscle pain	8	2.18%
Myalgia	**34**	**9.28%**
Arthromyalgia	8	2.18%
Arthralgia	11	3.00%
Muscular weakness	2	0.54%
Sleeplessness	**47**	**12.84%**
Dizziness	4	1.09%
Giddiness	2	0.54%
Depressed mood	7	1.91%
Weight gain	**45**	**12.29%**
Weight loss	**35**	**9.56%**
Total	366	100%

The five most commonly reported by patients are presented in bold.

**Table 4:** Relationships between Hospitalization Due to ADRs and Both Baseline and Average Medication Regimen Complexity Indices.

Baseline Medication Regimen Complexity Index				
Hospitalization due to ADR		0–8	>8	Total
	No	164	111	275
	Yes	2	8	10
Total		58.2%	41.8%	100.0%
**Average Medication Regimen Complexity Index**				
Hospitalization due to ADR		0–8	>8	Total
	No	161	114	275
	Yes	2	8	10
Total		57.1%	42.8%	100.0%

**Table 5:** Adverse drug reactions (ADRs) associated with hospitalization recorded at various follow-up visits.

Follow-up Visit Number	Number of Patients Hospitalized (N = 285%)	Causes for Hospitalization	Number of Days of Hospitalization Median (IQR)
1st	2 (22.22)	Pancytopenia Anemia, Nephrotic range proteinuria	6.5 (4–6.5)
2nd	1 (8.33)	Tenofovir-induced nephropathy	5 (5–5)
3rd	2 (15.38)	Iron Deficiency Anemia Possible Drug Reaction, Ornidazole, Cefixime, Amoxicillin	6 (4–6)
4th	2 (16.66)	Anemia and deficiencies in iron, vitamin B12, and folic acid Ethambutol-induced optic neuropathy	3 (2–3)
5th	0		Not applicable
6th	3 (37.5)	Anemia Zidovudine-induced macrocytic anemia Tenofovir-induced acute kidney injury (AKI)	4 (4–4)

(IQR 25th–50th)

**Table 6:** Follow-up Trends in Hospitalization Duration by Gender (n = 285).

Follow-Up Visit Number	Male	Female	First Episode of Hospitalization (n-%)	Second Episode of Hospitalization (n-%)	Number of Days of Hospitalization Median (IQR)
1st	7	2	9 (3.15)	1 (0.4)	3 (2–7)
2nd	8	4	12 (4.21)	0	5 (3–6)
3rd	9	4	13 (4.56)	0	4 (2–6)
4th	7	5	12 (4.21)	1 (0.4)	7 (3.75–8.25)
5th	3	4	7 (2.6)	0	4 (3–6)
6th	3	7	10 (3.7)	0	5 (3–8)

## Data Availability

The data generated and analyzed during the study are not publicly available due to privacy and ethical restrictions.
